# Development of Bifunctional Gadolinium-Labeled Superparamagnetic Nanoparticles (Gd-MnMEIO) for *In Vivo* MR Imaging of the Liver in an Animal Model

**DOI:** 10.1371/journal.pone.0148695

**Published:** 2016-02-17

**Authors:** Yu-Ting Kuo, Chiao-Yun Chen, Gin-Chung Liu, Yun-Ming Wang

**Affiliations:** 1 Department of Medical Imaging, Chi Mei Hospital, Tainan, Taiwan; 2 Department of Organ Transplantation Center, Kaohsiung Medical University Hospital, Kaohsiung, Taiwan; 3 Department of Medical Imaging, Kaohsiung Medical University Hospital, Kaohsiung, Taiwan; 4 Department of Radiology, Faculty of Medicine, College of Medicine, Kaohsiung Medical University, Kaohsiung, Taiwan; 5 Department of Biological Science and Technology, National Chiao Tung University, Hsinchu, Taiwan; 6 Department of Biomedical Science and Environmental Biology, Kaohsiung Medical University, Kaohsiung, Taiwan; University of South Florida, UNITED STATES

## Abstract

Liver tumors are common and imaging methods, particularly magnetic resonance imaging (MRI), play an important role in their non-invasive diagnosis. Previous studies have shown that detection of liver tumors can be improved by injection of two different MR contrast agents. Here, we developed a new contrast agent, Gd-manganese-doped magnetism-engineered iron oxide (Gd-MnMEIO), with enhancement effects on both T1- and T2-weighted MR images of the liver. A 3.0T clinical MR scanner equipped with transmit/receiver coil for mouse was used to obtain both T1-weighted spoiled gradient-echo and T2-weighted fast spin-echo axial images of the liver before and after intravenous contrast agent injection into Balb/c mice with and without tumors. After pre-contrast scanning, six mice per group were intravenously injected with 0.1 mmol/kg Gd-MnMEIO, or the control agents, i.e., Gd-DTPA or SPIO. The scanning time points for T1-weighted images were 0.5, 5, 10, 15, 20, 25, and 30 min after contrast administration. The post-enhanced T2-weighted images were then acquired immediately after T1-weighted acquisition. We found that T1-weighted images were positively enhanced by both Gd-DTPA and Gd-MnMEIO and negatively enhanced by SPIO. The enhancement by both Gd-DTPA and Gd-MnMEIO peaked at 0.5 min and gradually declined thereafter. Gd-MnMEIO (like Gd-DTPA) enhanced T1-weighted images and (like SPIO) T2-weighted images. Marked vascular enhancement was clearly visible on dynamic T1-weighted images with Gd-MnMEIO. In addition, the T2 signal was significantly decreased at 30 min after administration of Gd-MnMEIO. Whereas the effects of Gd-MnMEIO and SPIO on T2-weighted images were similar (p = 0.5824), those of Gd-MnMEIO and Gd-DTPA differed, with Gd-MnMEIO having a significant T2 contrast effect (p = 0.0086). Our study confirms the feasibility of synthesizing an MR contrast agent with both T1 and T2 shortening effects and using such an agent *in vivo*. This agent enables tumor detection and characterization in single liver MRI sections.

## Introduction

Liver disease is a major worldwide health problem, particularly in some developing countries [1, 2]. Chronic hepatitis B, hepatitis C, and alcoholic and non-alcoholic fatty liver diseases are endemic, prevalent [3], and the causes of chronic liver disease and liver cirrhosis. Furthermore, hepatocellular carcinoma (HCC) in patients with chronic liver disease or cirrhosis [4] or metastasis from extrahepatic primary malignancies, such as colorectal carcinoma [5] is associated with high mortality. Because early detection and diagnosis of liver tumors improve prognosis, great emphasis has been placed on developing methods to detect and diagnose liver tumors non-invasively. Tumor markers, such as α-fetoprotein (AFP), are relatively insensitive surveillance tools for the detection of chronic liver disease [6]. Imaging methods therefore play an even more important role in the management of patients with these liver diseases and at risk of liver cancer.

Of the various imaging methods, ultrasonography (US) is the most feasible and widely used. Combined with assessment of tumor markers such as AFP, B-mode US has been a well-established surveillance tool for patients at risk of HCC [7]. Practice guidelines recommend a US exam every six months to detect small HCCs in patients with chronic liver disease and at risk of developing HCC. If a suspicious lesion is detected on US, dynamic contrast-enhanced computed tomography (CT) or magnetic resonance imaging (MRI) is recommended to confirm lesion location and determine lesion characteristics. And according to the current consensus, HCC can be diagnosed based on typical CT or MRI findings in patients with liver cirrhosis of various etiology, or with chronic hepatitis B [8]. Although each has its own advantages and disadvantages, MRI according to a recent meta-analysis seems to be better than CT for diagnosing HCC [9]. However, compared to analysis of explanted liver (which is the gold standard method for detecting HCC lesions in cirrhotic liver), imaging methods (US, CT, and MRI) still have an unsatisfactory sensitivity (< 50%), particularly for detecting small (< 2 cm) lesions [10].

Most of the contrast agents used for CT or MRI are non-specific, extracellular agents, and excreted via the urinary system shortly after intravenous administration. Most of the agents used in MRI are gadolinium (Gd[III])-based and useful for T1-weighted sequences. On the other hand, other tissue-specific MR contrast agents, such as superparamagnetic iron-oxide (SPIO) compound (Ferucarbotran, Resovist, Bayer Healthcare, Berlin, Germany), which has been used to negatively enhance T2-weighted images, were reported to give better diagnostic performance with good sensitivity of lesion detection [11]. Yoo et al. maximized lesion detection by injecting both the Gd(III)-based T1 agent and SPIO T2 agent [12]. However, injection of two different contrast agents may be clinically impractical, and single MR contrast agents possessing both T1 and T2 shortening effects could potentially improve liver tumor detection on MRI. Although few such MR contrast agents have been developed [13–15], their potential usefulness in *in vivo* MR has been verified by local injection into normal animals [13] or subcutaneous tumor models [14]. The time course of contrast enhancement in liver by intravenous injection, the most common route of contrast administration, has not been previously evaluated.

The aim of this study was to develop a novel contrast agent with both T1 and T2 contrast effects on MR images of the liver and to test its value as a contrast agent *in vivo* in an animal model of liver cancer. In addition, intravenous administration and a clinical MR scanner were used to increase the translational value of the study.

## Materials and Methods

The details of the synthesis of methoxypoly(ethylene glycol) (mPEG)-acrylate (mPEG-AC), N-acryl-(3-aminopropyl)triethoxy silane (APTES-Ac), N,N’-APTES -N-Boc(ethylenediamine)-mPEG (mPEG-NBoc-silane), N,N’-APTES-N-Boc(ethylenediamine)-mPEG (mPEG-NH_2_-silane), and 1-(methyl-3-acetamidopropanoate)-4,7,10-tris(acetic acid)-1,4,7,10-tetraazacyclodecane (DOTA-COOH) are reported in the Supporting Information (S1 File).

### Synthesis of the MR contrast agent

#### Synthesis of MnMEIO nanoparticles

Iron acetylacetonate, manganese acetylacetonate, and benzyl ether were mixed and stirred under nitrogen. Oleic acid and oleylamine were then injected into the mixture, which was then heated to 200°C for 2 h, heated to 350°C for 1.5 h, cooled to room temperature, treated with acetone to precipitate MnMEIO nanoparticles, centrifuged (12,000 rpm) to remove solvent and unreacted reagents, and redispersed in chloroform to yield MnMEIO nanoparticles. The core diameter of the MnMEIO nanoparticles was confirmed by transmission electron microscopy (TEM).

#### Surface modification of the MnMEIO nanoparticles

The biological application of the MnMEIO nanoparticles was limited by the hydrophobicity of the oleic acid coating formed by high-temperature thermolysis. To increase the hydrophilicity of MnMEIO nanoparticles, their surfaces were modified by amino acid polymers. Oleic acid-coated MnMEIO nanoparticles (30 mg/mL) were dissolved in toluene (50 mL), treated with the hydrophilic polymer (mPEG-NH_2_-silane), heated to 60°C for 8 h, treated with hexane to precipitate the water soluble MnMEIO nanoparticles, dried under vacuum to remove hexane, and dispersed in water. The product was filtered through a 0.22-μm nylon filter and dialyzed using a dialysis membrane (M.W. cutoff = 50 kDa) for 24 h.

#### Synthesis of Gd-MnMEIO

1-Ethyl-3-(3-dimethylaminopropyl)carbodiimide hydrochloride (EDC; 1.55 mg, 10 mmol), and N-hydroxysuccinimide (NHS; 1.15 mg, 10 mmol) were added to a solution of Gd-DO3A-COOH (1 mL, 10 mM) and stirred at 600 rpm for 20 min. Then, the water soluble MnMEIO nanoparticles (10 mM) were added with stirring at 600 rpm for 90 min, and then filtered through a dialysis membrane (M.W. cutoff = 1 kDa) to remove unconjugated Gd-DO3A-COOH, EDC, and NHS.

### Animal model

The protocols for the animal experiments of the study have been approved by the Institutional Animal Care and Use Committee (IACUC) of Kaohsiung Medical University, Kaohsiung, Taiwan (Approval number: 99066). The CT26 murine colon carcinoma cells were grown in Dulbecco's minimal essential medium (DMEM) (Sigma, St Louis, MO, USA) supplemented with 5% fetal bovine serum (inactivated by heating at 56°C in a water bath for 30 min), 100 U/ml penicillin, and 100 μg/ml streptomycin, and incubated at 37°C in an atmosphere of 5% CO_2_. Eight-week-old Balb/c mice were purchased from the National Laboratory Animal Center of Taiwan (Taipei, Taiwan). All mice were raised in the laboratory animal center of our institution and were monitored by trained veterinary technicians every day to ensure their continued good health and welfare. They were raised in cages specific for experimental mice (six mice per cage). The size of each cage was 431.25in^3^ (L x W x H, 11.5 x 7.5 x 5.0 in). All the animal experiments were performed in accordance with institutional (Kaohsiung Medical University, Kaohsiung, Taiwan) guidelines. Animals were allowed *ad libitum* access to standard chow and drinking water. For the tumor model, about 2x10^6^ tumor cells were implanted subcutaneously into one of the hind limbs. After 2 weeks, the tumor grew to 10–15mm size and was removed. A small piece (about 1–2 mm^3^) of tumor tissue was implanted in the liver of each anesthetized mice by an experienced veterinary surgeon. During the operation, the animals were anesthetized by intraperitoneal injection of 90mg of ketamine and 10 mg of xylazine per kilogram body weight. After the operation, the mice were spontaneously recovered in a clear warm recovery area. The laparotomy wound was sutured and carefully cared by a veterinary surgeon. Antibiotic (Enrofloxacin [10 mg/kg daily], Baytril, Bayer Animal Health) was administered subcutaneously (SC) during the operation and for 3 days after surgery. Analgesics (Carprofen [1mg/kg] or Meloxicam [0.5mg/kg]) were also administered SC if animals were found to have decreased appetite or abnormal activity during recovery. About 7 days after tumor implantation, *in vivo* MRI was performed. The animal center, operating room, and MRI laboratory were in different buildings. The mice were acclimatized for 24 h before the operation and before MRI scanning, and euthanized by cervical dislocation after the experiment.

### *In vivo* MR imaging

Both T1- and T2-weighted images of liver were obtained in Balb/c mice with and without liver tumors before and after contrast administration. Each mouse was anesthetized with 80 mg/kg ketamine and 8 mg/kg xylazine, and placed in an animal coil in the prone position. The animal’s body temperature was maintained at 37°C by an automatic feed-back heating system throughout the period of MR data acquisition. MR imaging was performed using a clinical MR scanner (Signa HDxt, 3.0T; GE Healthcare, Milwaukee, MI; software version 15M4A 15A_M4A_0910) equipped with a home-made customized transmit/receiver volume coil for mouse (127.77MHz). T2-weighted coronal fat-saturated fast spin-echo (FSE)(TR/TE 3000/30 ms), T1-weighted spoiled gradient-echo (SPGR) (TR/TE/FA 800/10.6ms/12°), and T2-weighted (TR/TE 4000/34 ms) fat-saturated FSE axial images were obtained before and after intravenous injection of the contrast agent. The other scanning parameters for axial MR images were: field of view (FoV), 6x6cm; matrix, 256x192 for T2-weighted, 256x128 for T1-weighted; slice thickness, 3mm; number of excitations, 3 for for T2-weighted, 4 for T1-weighted. After pre-contrast scanning, six mice were intravenously injected with 0.1 mmol/kg of the newly synthesized agent (Gd-MnMEIO) and control agents, i.e., Gd-DTPA (Magnevist, Bayer Healthcare, Berlin, Germany) and SPIO (Resovist, Bayer Healthcare, Berlin, Germany). The scanning time points for the T1-weighted images were 0.5, 5, 10, 15, 20, 25, and 30 min after intravenous contrast administration. The post-enhanced T2-weighted axial images were then acquired immediately after post-contrast T1-weighted image acquisition.

### Imaging analysis and statistics

The operator-defined regions of interest (ROI) were placed on the normal liver and enhanced parts of the tumor using a hospital's picture archiving and communication system (UniSight, EBM Technologies, Taipei, Taiwan). Three ROIs for the liver and two ROIs for the tumor were measured. The ROIs were selected to avoid visible vascular structures and imaging artifacts. Images with significant motion artifacts in some mice were excluded from analysis. Mean signal intensities of the liver and tumor at the pre-contrast and post-contrast time points on both the T1- and T2-weighted images were then calculated. The enhancement percentage was calculated as:

$$\text{Enhancement}\;\left(\%\right)=\left[\left({\text{SI}}_{\text{t}}–{\text{SI}}_{\text{pre}}\right)/\;{\text{SI}}_{\text{pre}}\right]\times 100$$

where SI_t_ was the signal intensities of the ROIs at different post-contrast time points and SI_pre_ was the signal intensity at the pre-contrast time point.

In each group, the enhancement percentages at different time points were compared. Results are expressed as the mean ± standard deviation. A statistical software (Prism 6, GraphPad Software Inc., La Jolla, CA) was used to perform all statistical analyses. The two-way analysis of variance (ANOVA) random effects model and Student *t* test were used to compare between-group differences. Post-hoc comparison for enhancement at each time point was also carried out when a significant difference occurred. All p-values of less than 0.05 were considered statistically significant.

## Results

### Characterization of MnMEIO nanoparticles

#### The average particle size as obtained by transmission electron microscopy (TEM)

**Fig 1:**
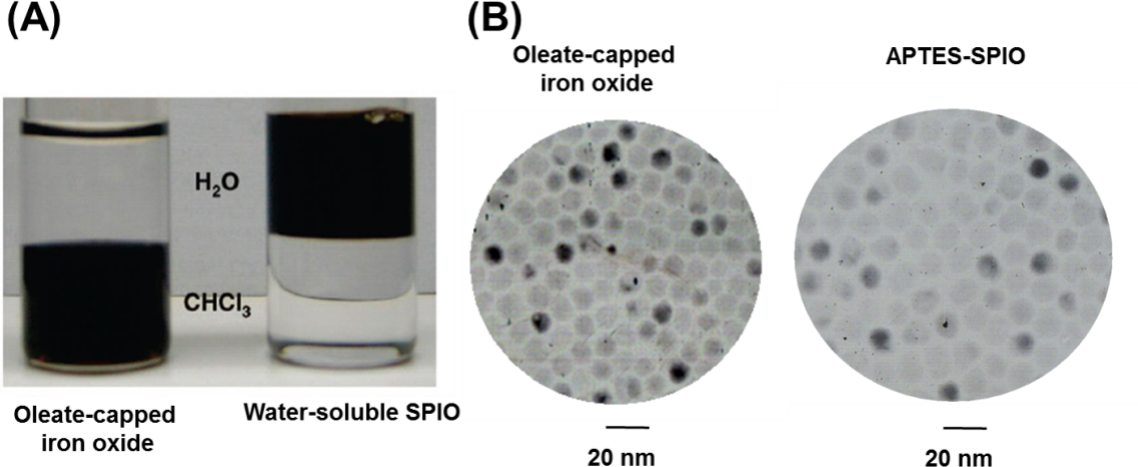
Manganese-doped magnetism-engineered iron oxide (MnMEIO) synthesized by thermal decomposition. (A) After modification with mPEG-NH_2_-silane, MnMEIO particles become water-soluble. (B) TEM images show that MnMEIO nanoparticles are around 12 nm in diameter with a narrow size distribution. Scale bar: 20 nm.

Manganese-doped magnetism-engineered iron oxide (MnMEIO) nanoparticles were produced by thermal decomposition. The surfaces of MnMEIO nanoparticles were modified by mPEG to increase their stability in aqueous suspension, as shown in Fig 1A. Analysis of TEM images revealed that the average core diameter of MnMEIO nanoparticles was 12.0 ± 2.0 nm and indicated that the mPEG-modified nanoparticles were well-dispersed (Fig 1B).

#### Other characteristics of Gd-MnMEIO nanoparticles

Dynamic light scattering (DLS) is a technique that can be used to determine the size distribution profile of small particles in suspension. A laser is shot through a sample and the scattered light then goes through a second polarizer where it is collected by a photomultiplier. The resulting image is projected onto a screen and used to determine average particle size and size distribution.

**Fig 2:**
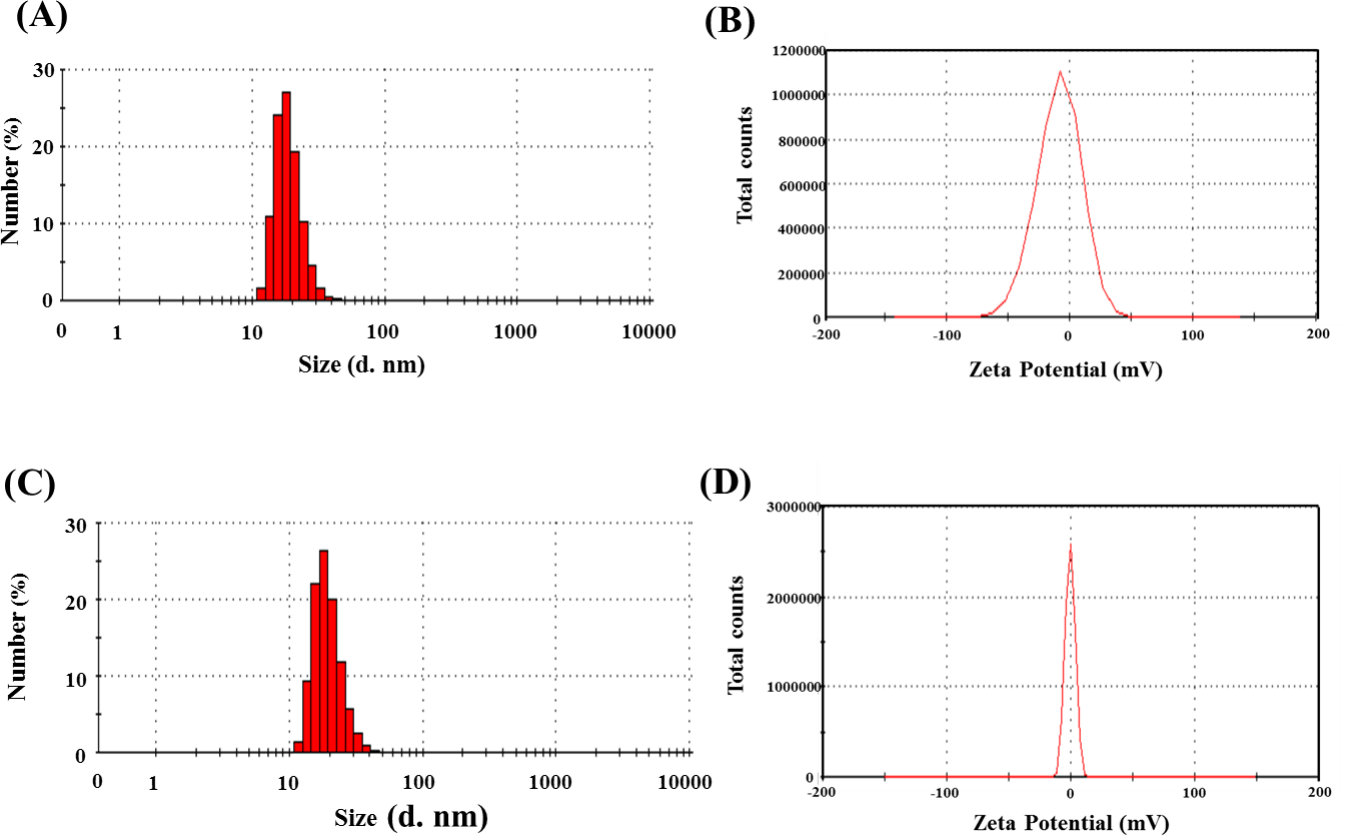
Dynamic light scattering (DLS) and zeta potential analysis of the MnMEIO-PEG (A) and Gd-MnMEIO (B) particles.

Polyethylene glycol (PEG) was used to prepare coated MnMEIO particles through a ligand-exchange reaction. DLS analysis was carried out after the ligand-exchange reaction to determine the hydrodynamic size of MnMEIO-PEG. As shown in Fig 2A, the average size and zeta potential of MnMEIO-PEG nanoparticles were about 19.0 ± 4.5 nm and –0.88 ± 4.7 mV. The conjugation of Gd(III) with MnMEIO-PEG nanoparticles had little effect on the hydrodynamic size of MnMEIO-PEG nanoparticles (Fig 2B), indicating that Gd(III) complexes were too small to affect the average size of Gd-MnMEIO nanoparticles (which was 19.6 ± 5.1 nm). Moreover, the zeta potential decreased to –10.34 mV, probably because of the carboxyl groups of Gd(III) complexes conjugated with the MnMEIO-PEG nanoparticles.

The contrast efficiency of MRI contrast agents was affected by the amount of Gd(III) in Gd-MnMEIO. Analysis of Gd-MnMEIO components by inductively coupled plasma atomic absorption spectrometry (ICP-AAS) found iron, manganese, and gadolinium in a ratio of 100:4:1. Using a 20-MHz relaxometer, MnMEIO was found to have relaxivity values (*r*_1_ and *r*_2_) of 39.6 mM^–1^s^–1^ and 171.9 mM^–1^s^–1^, respectively (and the ratio of *r*_2_/ *r*_1_ was 4.3) (Figure G in S1 File). However, the *r*_1_ and *r*_2_ values of Gd-MnMEIO were 60.8 mM^–1^s^–1^ and 149.9 mM^–1^s^–1^, respectively (and the ratio of *r*_2_/ *r*_1_ was decreased to 2.5). The large increase in longitudinal relaxivity was attributed to the addition of gadolinium(III) complexes to MnMEIO.

### *In vivo* imaging

**Fig 3:**
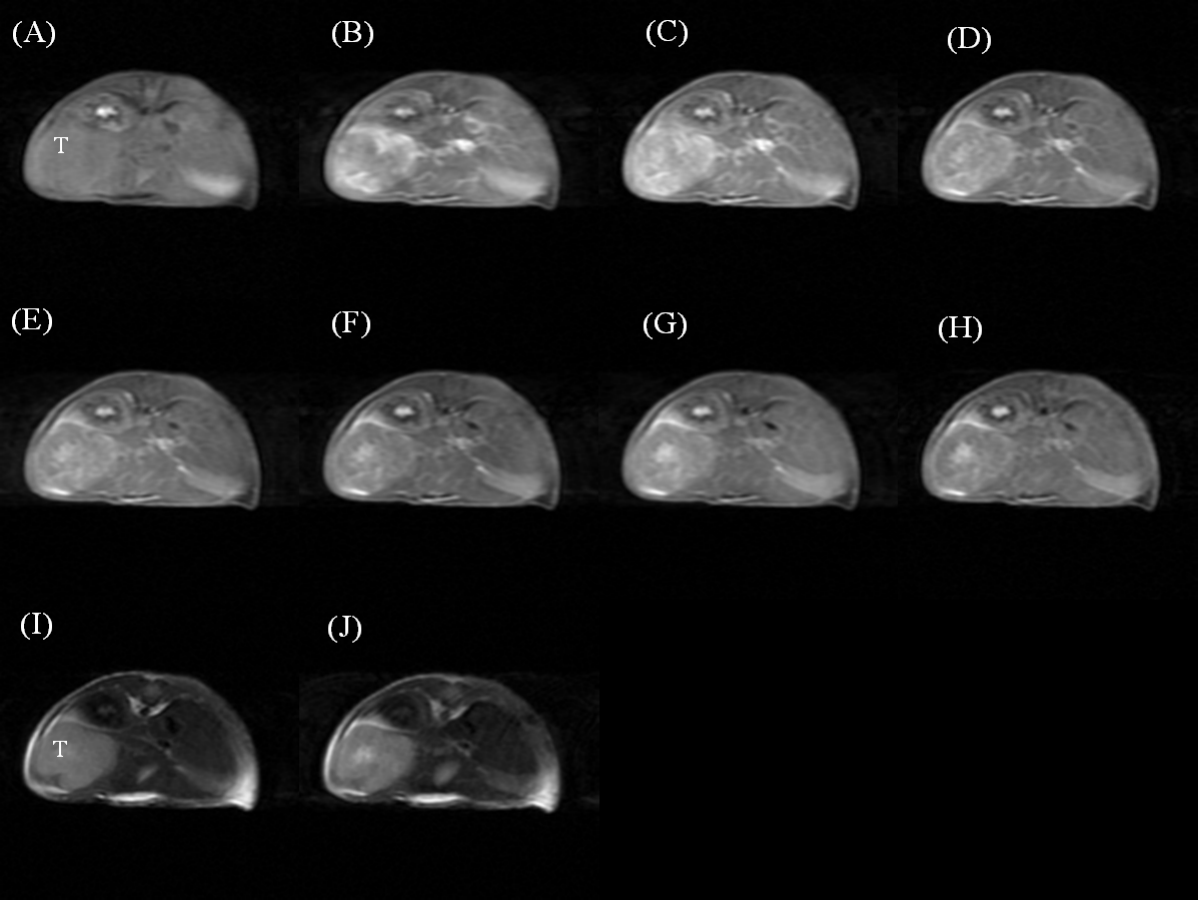
Dynamic contrast enhanced T1-weighted images before (A), 0.5 (B), 5 (C), 10 (D), 15 (E), 20 (F), 25 (G), and 30 (H) min after intravenous administration of Gd-DTPA. And T2-weighted images before (I) and 30 min after (J) Gd-DTPA administration. T, tumor.

**Fig 4:**
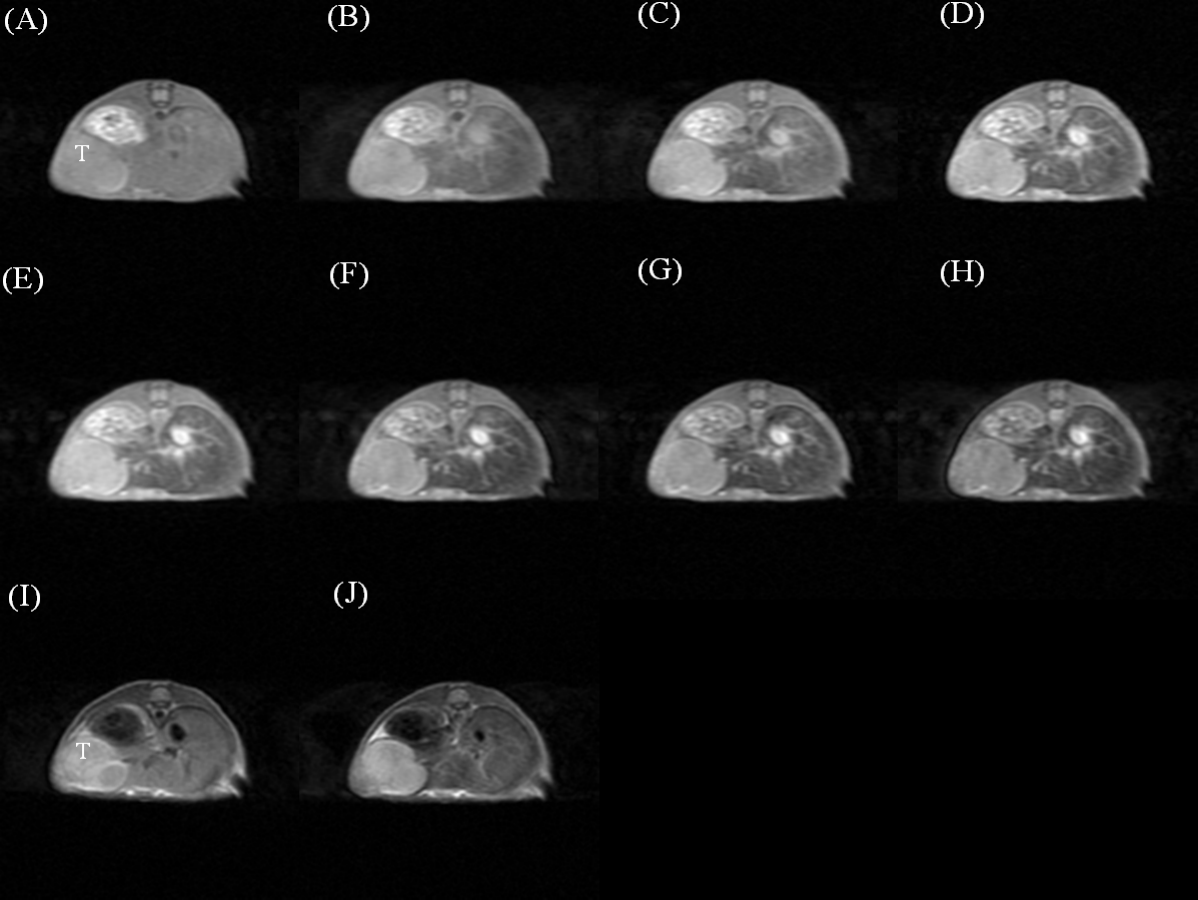
Dynamic contrast enhanced T1-weighted images before (A), 0.5 (B), 5 (C), 10 (D), 15 (E), 20 (F), 25 (G), and 30 (H) min after intravenous administration of SPIO. And T2-weighted images before (I) and 30 min after (J) SPIO administration. T, tumor.

**Fig 5:**
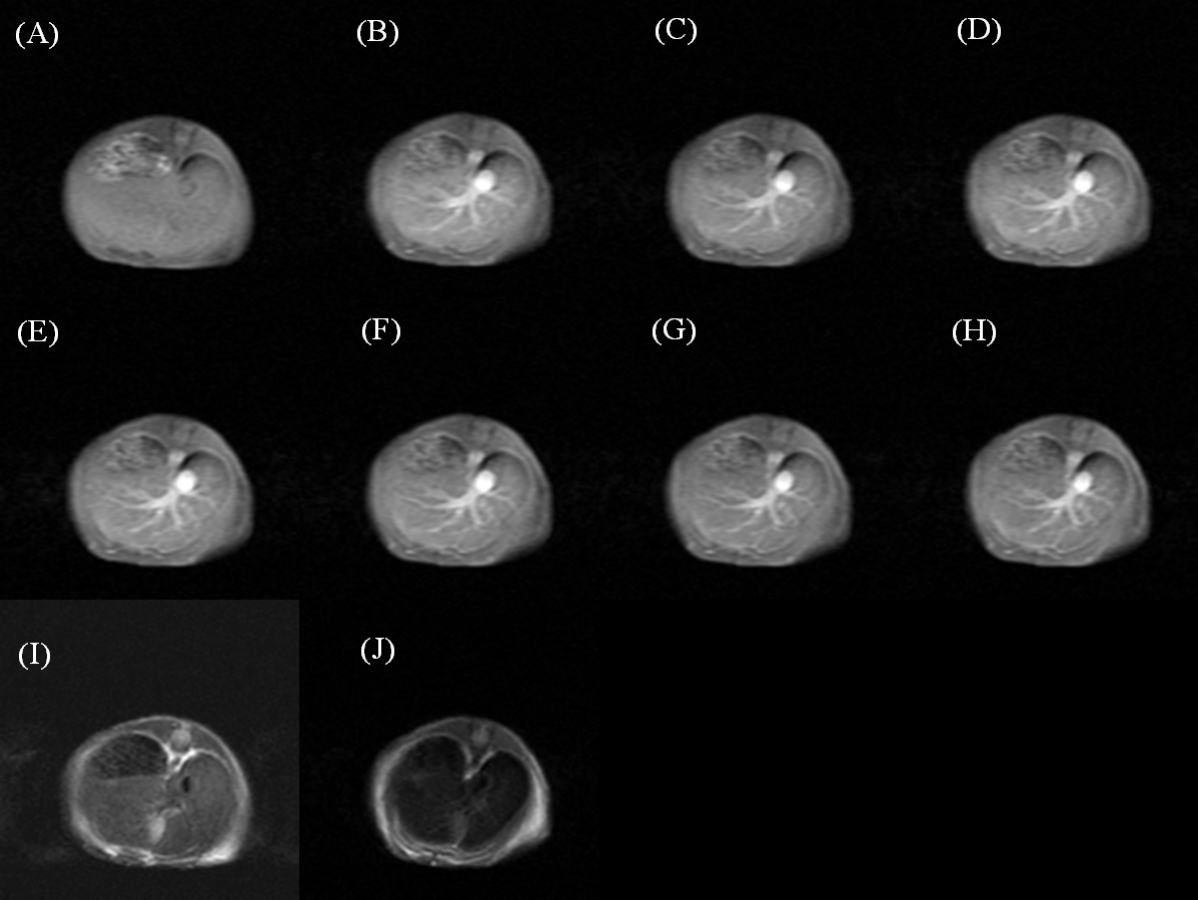
Dynamic contrast enhanced T1-weighted images before (A), 0.5 (B), 5 (C), 10 (D), 15 (E), 20 (F), 25 (G), and 30 (H) min after intravenous administration of Gd-MnMEIO. And T2-weighted images before (I) and 30 min after (J) Gd-MnMEIO administration.

**Fig 6:**
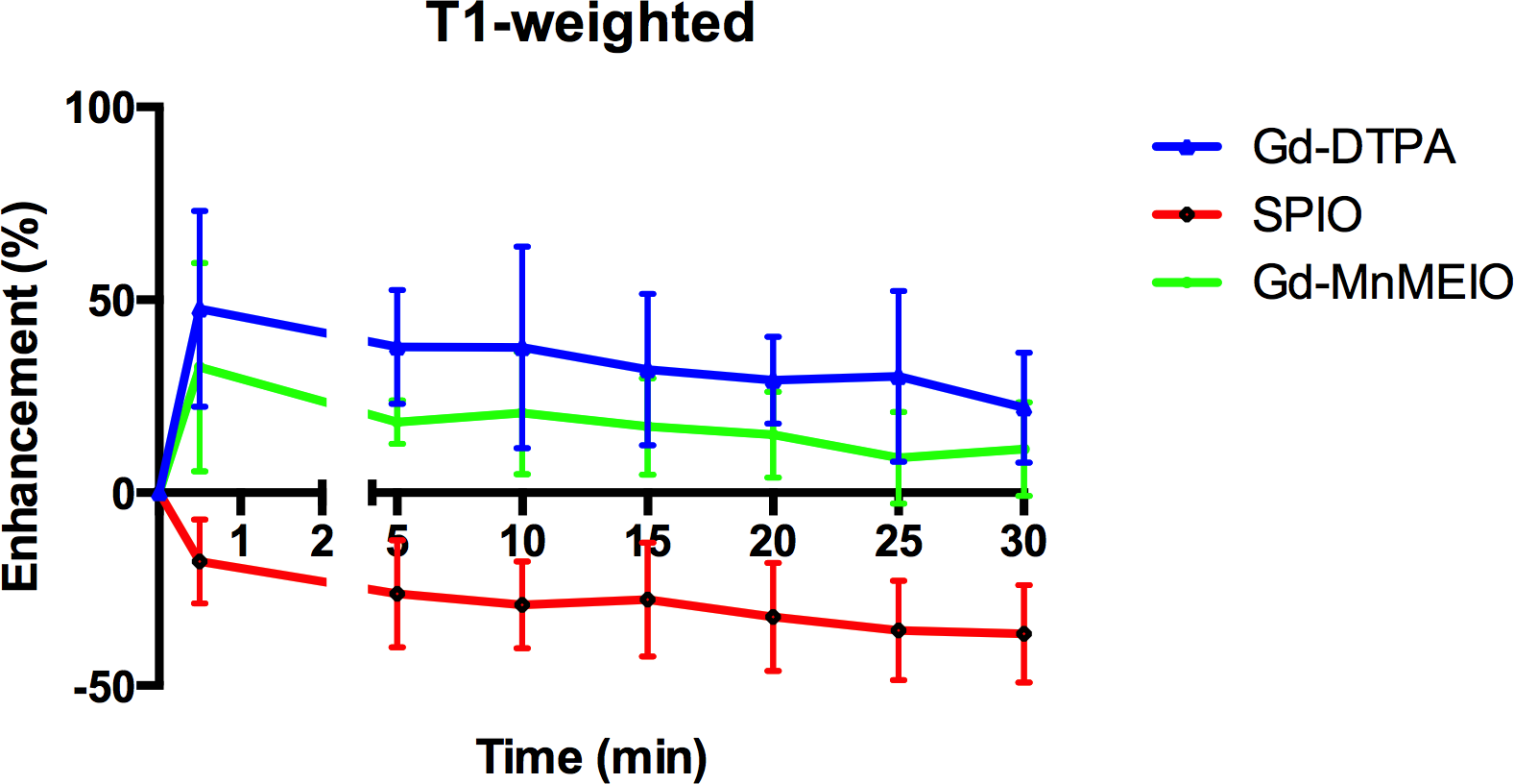
Time course curves of liver parenchyma enhancement on T1-weighted images by using Gd-DTPA (n = 8), SPIO (n = 8), and Gd-MnMEIO (n = 8). The error bars indicate standard deviation. Contrast enhancement with Gd-DTPA and Gd-MnMEIO is significantly better than with SPIO at every post-enhancement time point (p <0.001), while no significant difference can be observed between Gd-DTPA and Gd-MnMEIO (p > 0.05).

**Fig 7:**
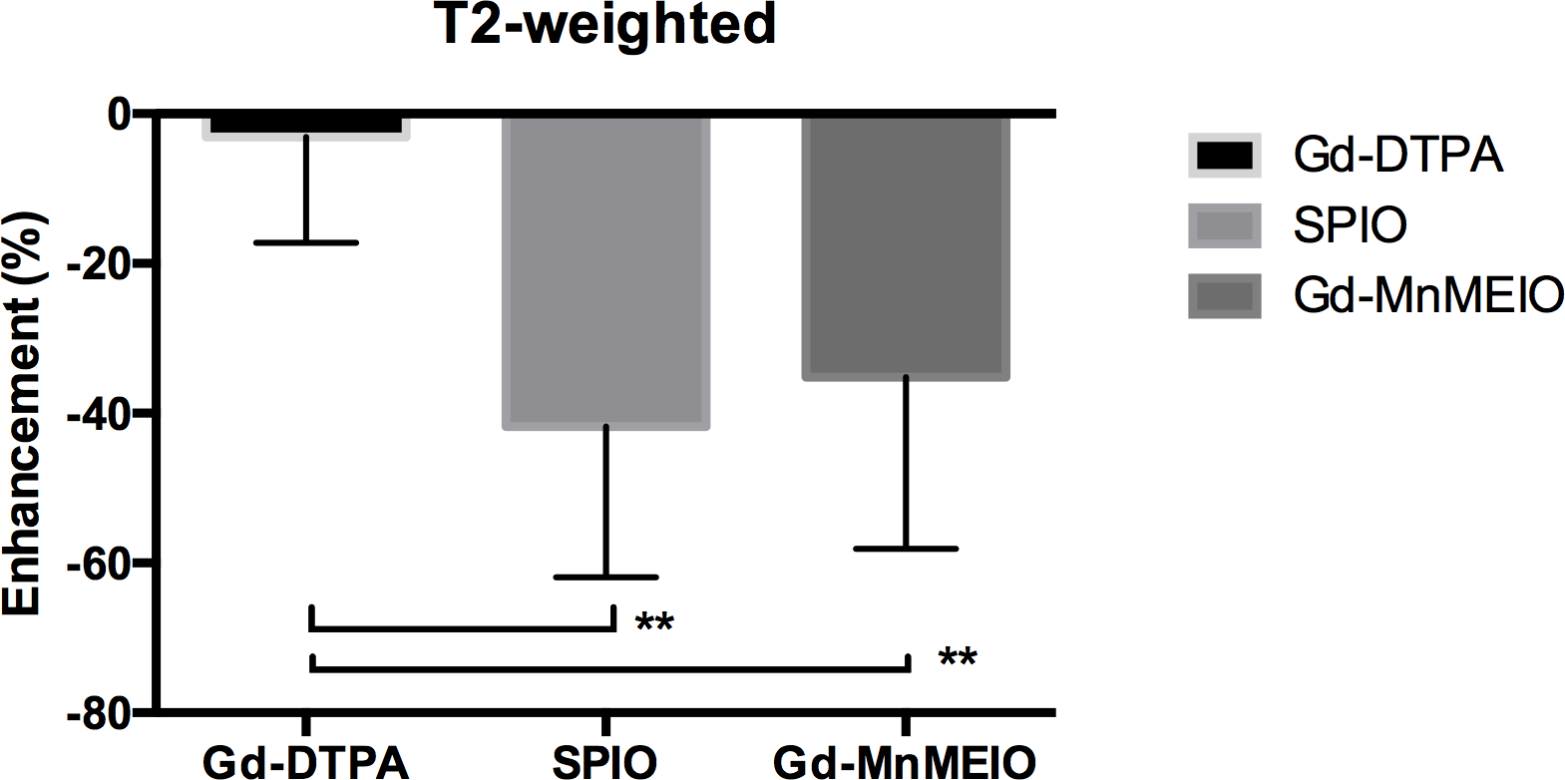
T2 enhancement effect at 30 min after administration of Gd-DTPA (n = 9), SPIO (n = 15), and Gd-MnMEIO (n = 6). The error bars indicate standard deviation. Significant difference is indicated by ** p < 0.01.

The dynamic contrast-enhanced T1- and T2-weighted images of a mouse injected with Gd-DTPA (Fig 3), SPIO (Fig 4), and Gd-MnMEIO (Fig 5) are presented. On T1-weighted images, both Gd-DTPA and Gd-MnMEIO yields positive contrast enhancement, while SPIO provided negative contrast enhancement (Fig 6). Both the enhancement due to Gd-DTPA and Gd-MnMEIO peaked at 0.5 min and then decreased gradually thereafter. However, Gd-MnMEIO (like Gd-DTPA) had significant T1 enhancement effects and (like SPIO) significant T2 enhancement effects (Figs 6 and 7). Significant major vascular enhancement was clearly observed on the dynamic T1-weighted images with Gd-MnMEIO (Fig 5B–5H). In addition, the T2 signal was significantly decreased at 30 min after administration of Gd-MnMEIO (Fig 5J). The Gd-DTPA provided better T1 enhancement than Gd-MnMEIO (p = 0.0289) and SPIO (p < 0.0001). On the other hand, SPIO had the greatest signal-reduction effect on post-enhanced T2-weighted images. There was no significant difference in the T2 contrast effect between Gd-MnMEIO and SPIO (p = 0.5824). Compared with Gd-DTPA (which had no T2 shortening effect), Gd-MnMEIO had a significantly greater T2 contrast effect (p = 0.0086) (Fig 7).

**Fig 8:**
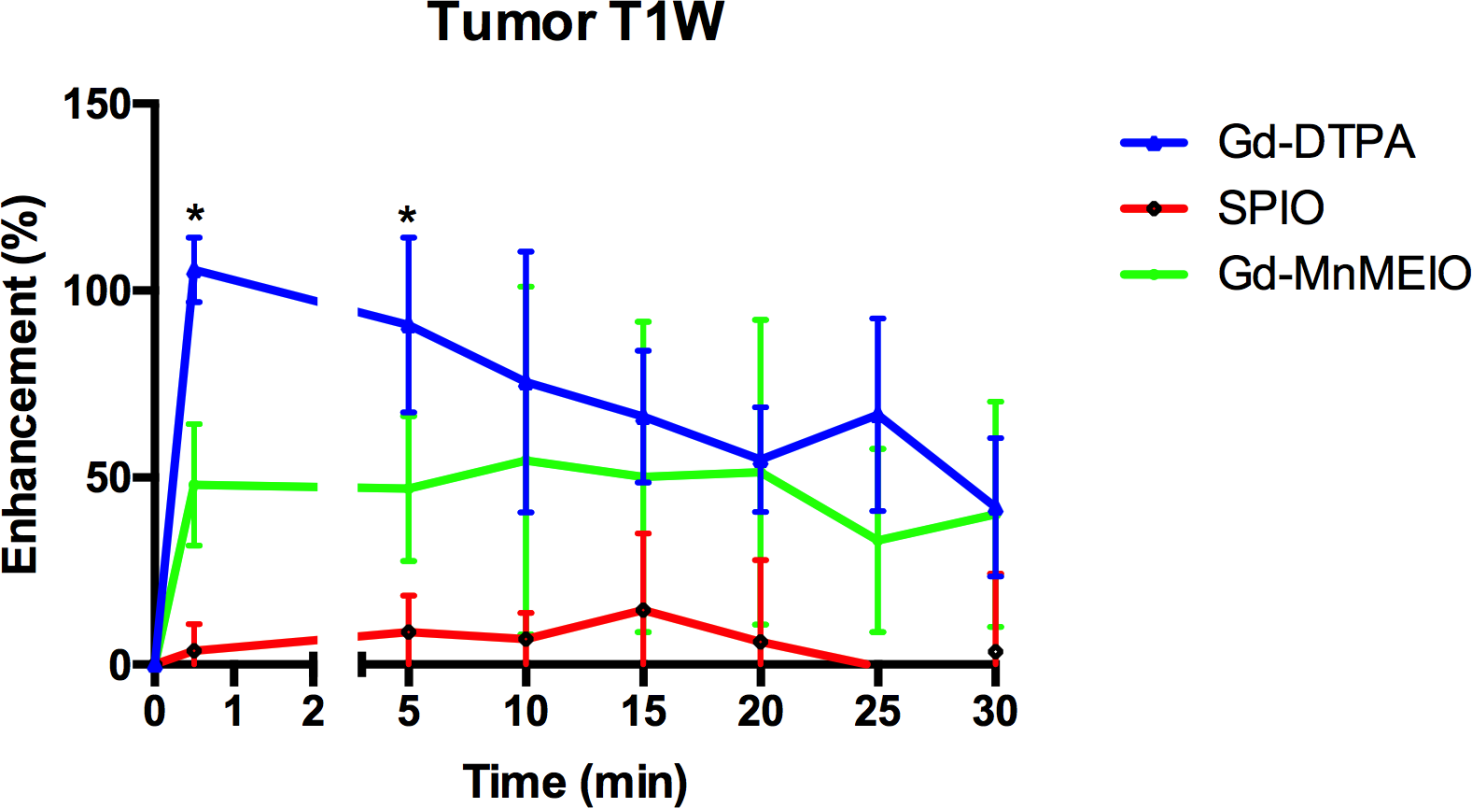
Time course curves of implanted liver tumor enhancement on T1-weighted images by using Gd-DTPA (n = 4), SPIO (n = 7), and Gd-MnMEIO (n = 5). The error bars indicate standard deviation. Significant differences are indicated by * (p < 0.05 between Gd-DTPA and Gd-MnMEIO, p < 0.01 between Gd-MnMEIO and SPIO).

**Fig 9:**
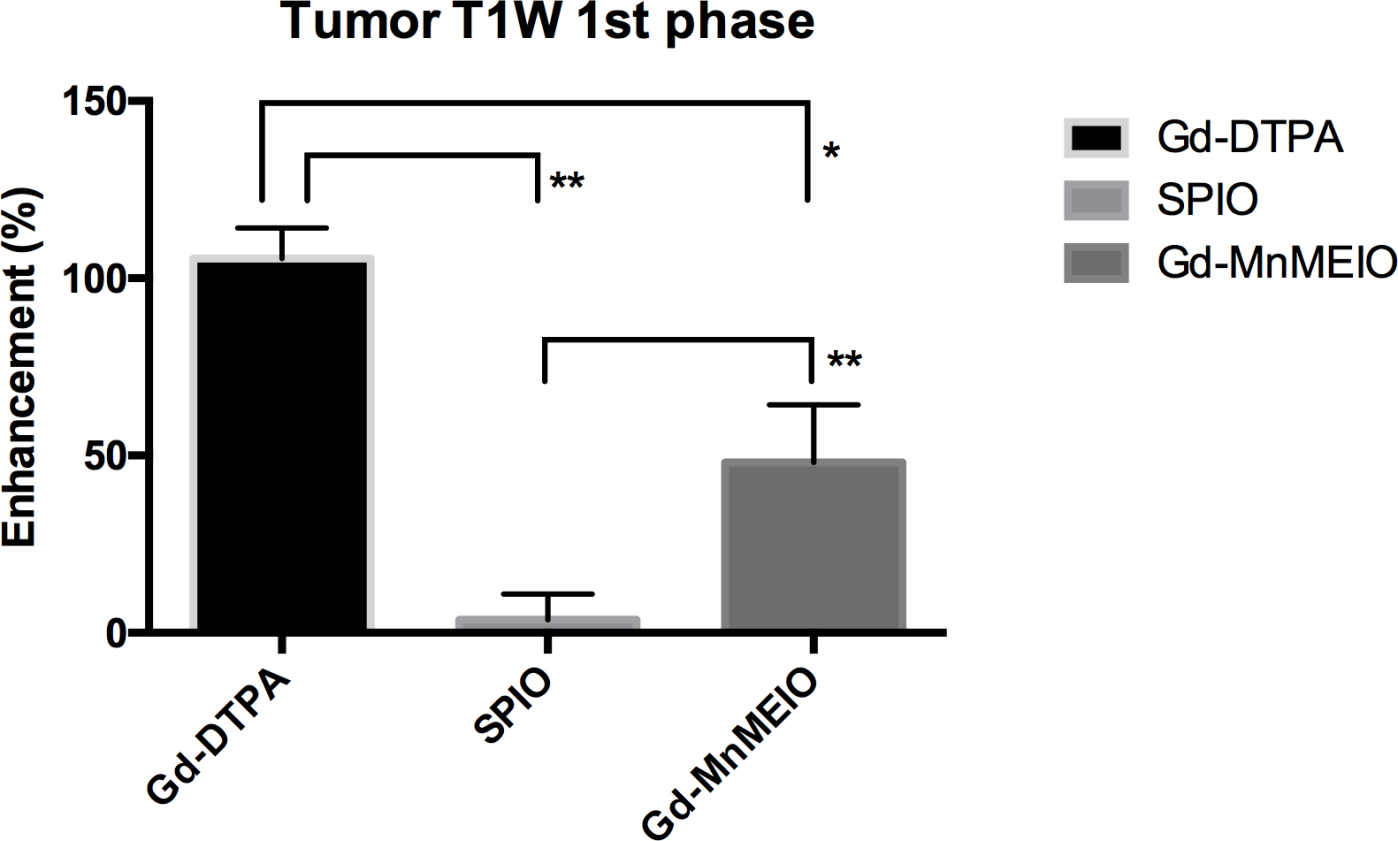
T1 enhancement of tumor in the first phase of dynamic contrast-enhanced T1-weighted images with Gd-DTPA (n = 4), SPIO (n = 7), and Gd-MnMEIO (n = 5). The error bars indicate standard deviation. Significant differences are indicated by * p < 0.05 and ** p < 0.01.

Gd-DTPA and Gd-MnMEIO but not SPIO had strong enhancement effects on visualization of the tumors seen in dynamic T1-weighted images (Fig 8). Tumor enhancement with Gd-DTPA or Gd-MnMEIO peaked early after injection, was significantly more intense with Gd-DTPA than Gd-MnMEIO on early phase dynamic T1-weighted images (p = 0.0159) (Fig 9), but was similar in late phase T1-weighted images.

**Fig 10:**
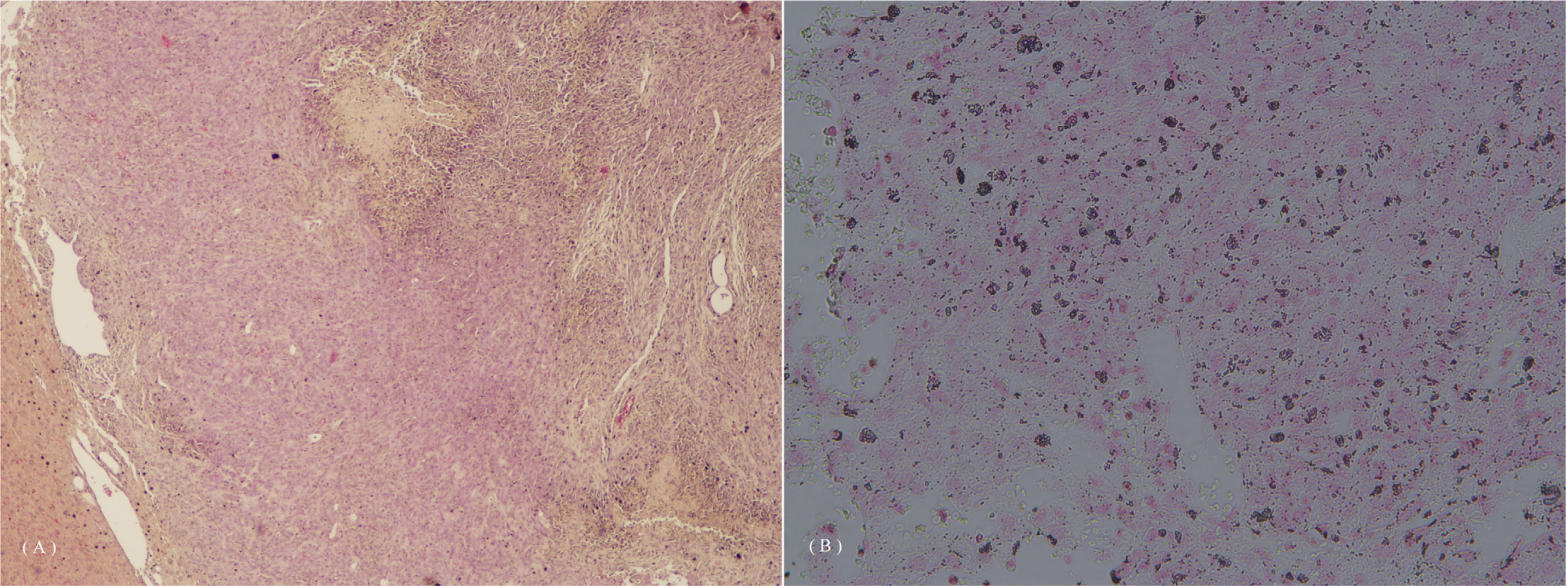
Liver pathology in the mouse after intravenous Gd-MnMEIO administration showed deposition of the contrast agent. (A) Hematoxylin and eosin staining, HE staining; (B) Prussian blue staining, PB staining.

The deposition of the injected contrast agent, as observed by microscopy (Fig 10A and 10B), showed that the signal intensity changes in the liver and tumor were due to contrast agent administration.

## Discussion

The method of Gd-MnMEIO synthesis (in which EDC-NHS mediates conjugation between the amine groups of MnMEIO and carboxylic groups of the Gd[III] complex) used in our study differs from methods used in previous studies [13, 14]. Fewer steps were required for the synthesis of Gd-MnMEIO nanoparticle synthesis in our study than for the mPEG-DOPA (dopamine-modified iron oxide nanoparticles and Gd-DTPA by amine groups of dopamine and cyanides of DTPA) in another study [13]. The core-shell synthesis that combined both materials was also developed in one of previous studies [14]. Our Gd-MnMEIO nanoparticles had a high average hydrodynamic size of 20 nm and zeta potential of near 0 mV, which effectively prevented their clearance from the reticuloendothelial system (RES) and increased their retention time [16, 17]. Furthermore, the relaxivity *r*_1_ and *r*_2_ values of Gd-MnMEIO were extremely high compared to those of commercially available contrast agents [18].

Our *in vivo* imaging results clearly demonstrate that Gd-MnMEIO can not only enhances normal liver and viable tumor tissues but also improves visualization of the vascular tree. To diagnose a liver tumor, the hemodynamic information obtained from dynamic contrast-enhanced T1-weighted MRI images is essential. Non-specific gadolinium-based contrast agents are currently used in clinical practice to differentiate liver tumors based on hemodynamic information acquired from MR images. To the best of our knowledge, this study is the first *in vivo* MR study using this group of contrast agents to perform standard dynamic scanning studies in an animal model. For some tumors without obvious vascularity, T2 contrast may provide an additional opportunity for lesion detection and characterization. Our study validates the feasibility of designing a contrast agent (i.e., a combination of gadolinium[III] complex and iron oxide nanoparticles) with two different contrast-enhancement effects.

In current clinical imaging practice, most contrast-enhanced MRI examinations are carried out as T1-weighted sequences with Gd-based contrast agents. The non-tissue-specific extracellular Gd-based agents provide good hemodynamic information on T1-weighted images. On the other hand, iron oxide nanoparticles, commercially available as T2 contrast agents, can serve as molecular imaging probes because of their susceptibility to phagocytosis by macrophages [19]. Normal or abnormal phagocytosis of iron oxide decreases the signal intensity of tissues on T2-weighted MR images. Some studies in humans have used these T2 agents to detect metastatic lymphadenopathy [20] and infection/inflammation processes [21]. However, the information revealed in images enhanced by most T2 contrast agents is merely supplemental to information obtained from images enhanced by T1 contrast agents. Therefore, combining the capabilities of both T1 and T2 agents into a single MR contrast agent may be expected to maximize the amount of clinical information available from a single MRI study.

The study has some limitations. First, the contrast enhancement in our tumor model (an implanted tumor) may differ from that in *de novo* lesions. Implanted liver tumors may consist of peripheral areas of viable tumor (which are assessable) and large areas of tumor necrosis (which are not assessable). Therefore, variation of signal intensity due to ‘contamination’ from adjacent liver parenchyma may be unavoidable. Second, further optimization of the agent is probably needed to improve both T1 and T2 contrast-enhancement effects. This could be achieved by increasing the T1 relaxivity of the contrast agent and increasing its uptake by hepatocytes, Kupffer cells, or tumor cells. Third, other T1-weighted sequences and scanning parameters (such as changing flip angles of gradient-echo sequences) can also be tested to maximize the contrast enhancement effect. Additionally, we could not compare the sensitivity or specificity of liver tumor detection between the synthesized agent and commercially-available clinical agents because of limitations imposed by the animal model and our experimental methods. *In vivo* imaging experiment on genetically-modified model of cancer which can be used to track *de novo* tumor progression in liver [22] may be needed for this purpose. Nevertheless, our study was a feasibility study which aimed to validate this concept. Further studies are mandatory if translation to clinical or pre-clinical use is to be achieved.

Another limitation is that our agent was not compared with other Gd(III)-based extracellular and hepatocyte-specific T1 contrast agents, i.e., Gd-BOPTA (Multihance, Bracco Diagnostics, Milan, Italy) and Gd-EOB-DTPA (Primovist, Bayer Healthcare, Berlin, Germany). This particular group of Gd-based agents has gained much attention in lesion detection and characterization [23] and may be used for problem-solving when standard dynamic contrast-enhanced CT or MRI with extracellular contrast agents yield indeterminate findings [24]. Hepatobiliary-phase T1-weighted images with this group of Gd-based agents were reported to be able to differentiate pre-malignant cirrhotic nodules from early HCC [25, 26]. Use of these agents also increased the sensitivity of lesion detection in HCC lesions after standard dynamic contrast-enhanced CT, thereby changing treatment options [27], but were associated with some technical problems of liver MRI (including specific imaging artifacts and problems with arterial phase timing). Most modern scanners require further manipulation to optimize imaging quality or newer imaging sequences [28–30]. This can hinder its widespread clinical application. Development of other kinds of contrast agents is therefore potentially useful.

In conclusion, our study confirms the feasibility of synthesis of an MR contrast agent with both T1 and T2 shortening effects. The uptake of this newly synthesized agent by liver tumor and normal liver tissues is validated by *in vivo* MRI study in normal mice and a mouse liver tumor model. We also demonstrated the feasibility of using this new agent for dynamic contrast-enhanced MRI, which is very important for liver tumor characterization. Endowing a single agent with two different imaging contrast effects may improve the possibility of liver tumor detection and characterization from a single MR imaging section. Despite its limitations, this kind of MR contrast agent should be further investigated and optimized to maximize its benefit in patients with chronic liver diseases, who are at risk for developing liver tumors.

## Supporting Information

S1 File Synthesis of MnMEIO nanoparticles. (PDF)
