# Supplementary material for: Development of Bifunctional Gadolinium-Labeled Superparamagnetic Nanoparticles (Gd-MnMEIO) for In Vivo MR Imaging of the Liver in an Animal Model
Source: PLoS One. 2016 Feb 17;11(2):e0148695. doi: 10.1371/journal.pone.0148695 (PMC4757547; doi:10.1371/journal.pone.0148695)
Supplement: S1 File — (PDF) [file pone.0148695.s001.pdf]

Supporting Information. Synthesis of MnMEIO nanoparticles

Synthesis of mPEG-acrylate (mPEG-Ac)

Methoxy poly(ethylene glycol) (mPEG) (20 g, 10 mmol) was dissolved in 150 mL of dry dichloromethane (DCM). After the solution was cooled to 0°C in an ice-water bath, acryloyl chloride (2.4 mL, 30 mmol) and triethylamine (TEA; 4.2 mL, 30 mmol) were added to the solution via syringe. The solution was stirred at room temperature for 48 h and rotary evaporated to remove the reaction solvent. The crude product was dissolved in tetrahydrofuran (THF) and the residue was discarded. The filtrate was precipitated in cold hexane and separated by draining. Finally, the solid product was dried under vacuum overnight (31.9 g, 76% yield). ^{1}H NMR (300 MHz, $CDCl_3$, d) (Fig A): 3.37 (s, 3H, CH_3-O), 3.63 (m, 104H, PEG chain protons), 4.30 (t, J = 4.7 Hz, 2H, C-CH_2-C=O), acryl group: 5.56 (1H), 6.15 (1H), 6.37 (1H).

Synthesis of N-acryl-(3-aminopropyl)triethoxy silane (APTES-Ac)

In a typical reaction, APTES (9.33 mL, 40 mmol) was dissolved in 200 mL of dry DCM. After the solution was cooled to 4°C, acryloyl chloride (4.60 g, 50 mmol) and TEA (12.9 mL, 80 mmol) were added to the solution via syringe. The solution was stirred at room temperature for 6 h. After the reaction, the solvent was evaporated under reduced pressure and the mixture was purified by silica-gel chromatography (hexane：ethyl acetate = 1：1) (7.9 g, 71.71% yield). ^{1}H NMR (300 MHz, $CDCl_3$, d) (Fig B)：0.66 (t, J = 7.1 Hz, 2H, C-CH_2-Si), 1.19 (q, J = 8.1 Hz, 9H, CH_3-CH_2-O), 1.65 (m, J = 7.5 Hz, 2H, C-CH_2-C), 3.32 (t, J = 7.1 Hz, 2H, C-CH_2-N), 3.81 (q, J = 8.4 Hz, 6H, C-CH_2-O), acryl group：5.61 (1H), 6.11 (1H), 6.21 (1H).

Synthesis of N,N'-APTES -N-Boc(ethylenediamine)-mPEG (mPEG-NBoc-silane)

In the typical synthesis, APTES-Ac (1.8 g, 6.6 mmol), mPEG-Ac (13.6 g, 6.6 mmol), and N-Boc ethylenediamine (0.8 g, 7.0 mmol) were dissolved in 20 ml of dry DCM. Subsequently, TEA (0.8 ml, 7.0 mmol) was transferred into the flask via syringe and the resulting solution was stirred for 72 h at 40–45°C under nitrogen. The polymerization was terminated by cooling the reaction down to room temperature. The product was precipitated in cold hexane, separated by draining, and dried under vacuum overnight (4.5 g, 39.4% yield). ^{1}H-NMR (300 MHz, $CDCl_3$, d) (Fig C)：0.76 (t, 2H, Si-CH_2-C, J = 4.2 Hz), 1.24 (t, 9H, CH_2-CH_3, J = 2.4 Hz), 1.42 (s, 9H, C-CH_3), 2.48-2.67 (m, 2H, -C-CH_2-(C=O)-N, 2H, -C-CH_2-(C=O)-O), 2.82-2.98 (m, 2H, N-CH_2-C-(C=O)-O; 2H, N-CH_2-C-(C=O)-N, 2H, N-CH_2-C-N), 3.20 (t, 2H, -C-CH_2-NH-(C=O)), 3.27 (s, 3H, -O-CH_3), 3.54-3.70 (m, ~190H, -(CH_2-O-CH_2)n-), 4.18 (t, 2H, C-CH_2-O-(C=O), J = 4.2 Hz).

Synthesis of N,N'-APTES -N-Boc(ethylenediamine)-mPEG (mPEG-NH_2-silane)

Silane-*N*-Boc-EA-mPEG (4.5 g, 1.79 mmol) was dissolved in 10 mL of dichloromethane in a round-bottom flask, followed by addition of 0.6 mL of trifluoroacetic acid (TFA). The solution was stirred for 6 h at room temperature. The final product, silane-ethylamine-methoxy poly(ethylene glycol) (silane-EA-mPEG), was precipitated in ethyl ether : hexane = 1 : 1, separated by draining, and dried under vacuum overnight (3.23 g, 78% yield). ^{1}H-NMR (300 MHz, $CDCl_3$, δ) (Fig D)：2.54 (m, 2H, -C-CH_2-(C=O)-N-, 2H, -C-CH_2-(C=O)-O-), 2.67 (m, 2H, N-CH_2-C-(C=O)-N-, 2H, N-

CH_2-C-NH_2), 2.86 (m, 2H, N-CH_2-C-(C=O)-O-, 2H, N-C-CH_2-NH_2), 3.13 (q, 2H, -C-CH_2-NH-(C=O), 3.32 (s, 3H, -O-CH_3), 3.59-3.70 (m, ~190H, -(CH_2-O-CH_2)n-), 4.22 (t, 2H, C-CH_2-O-(C=O), J = 7.8 Hz), 4.45 (s, 3H, Si-OH).

Synthesis of 1-(methyl 3-acetamidopropanoate)-4,7,10-tris(acetic acid)-1,4,7,10-tetraazacyclodecane (DO3A-COOH)

DO3A-tris-tbutyl ester (2 g, 3.89 mmol) was dissolved in 80 mL of CH_3CN. At the same time, K_2CO_3 (0.54 g, 3.89 mmol) and methyl 3-(2-bromoacetamido) propanoate (0.87 g, 3.89 mmol) were added to the solution, and stirred and refluxed at 4°C for 24 h. After the reaction was completed, the solvent was vaporized under reduced pressure. The residue was dissolved in H_2O, and extracted by DCM. The organic layer was treated with $MgSO_4$ to remove water. The solution was dried under reduced pressure to yield yellow crude product. Then the crude product was dissolved in 100 mL of 6N HCl, and stirred at room temperature for 16 h. The solution was vaporized under reduced pressure, and the pH was adjusted to 11 using H_2O. The crude product was further purified by anion exchange chromatography to yield a white powder (1.25 g, 67.6% yield). ^{1}H-NMR (300 MHz, D_2O, δ) (Fig E)：2.58 (t, 2H), 3.07-3.81 (m, 26H).

Synthesis of Gd(DO3A-COOH)

As shown in Scheme, DO3A-COOH (100 mg, 0.21 mmol) was dissolved in 5 mL of H_2O, and the pH was adjusted to 6 using 1N NaOH. $GdCl_{3(aq)}$ (0.99 eq) was then added to the above solutions and stirred at room temperature for 72 h. After the reaction was completed, Chelex-100 was added and stirred for 10 min. The solution was filtered through a 200-nm nylon filter, and lyophilized to

give a white powder (122 mg, 92% yield). The structure was determined by ESI-MS (m/z): calculated for $[M+H]^+$, 629.7, found 630.9. (Fig F).

Scheme

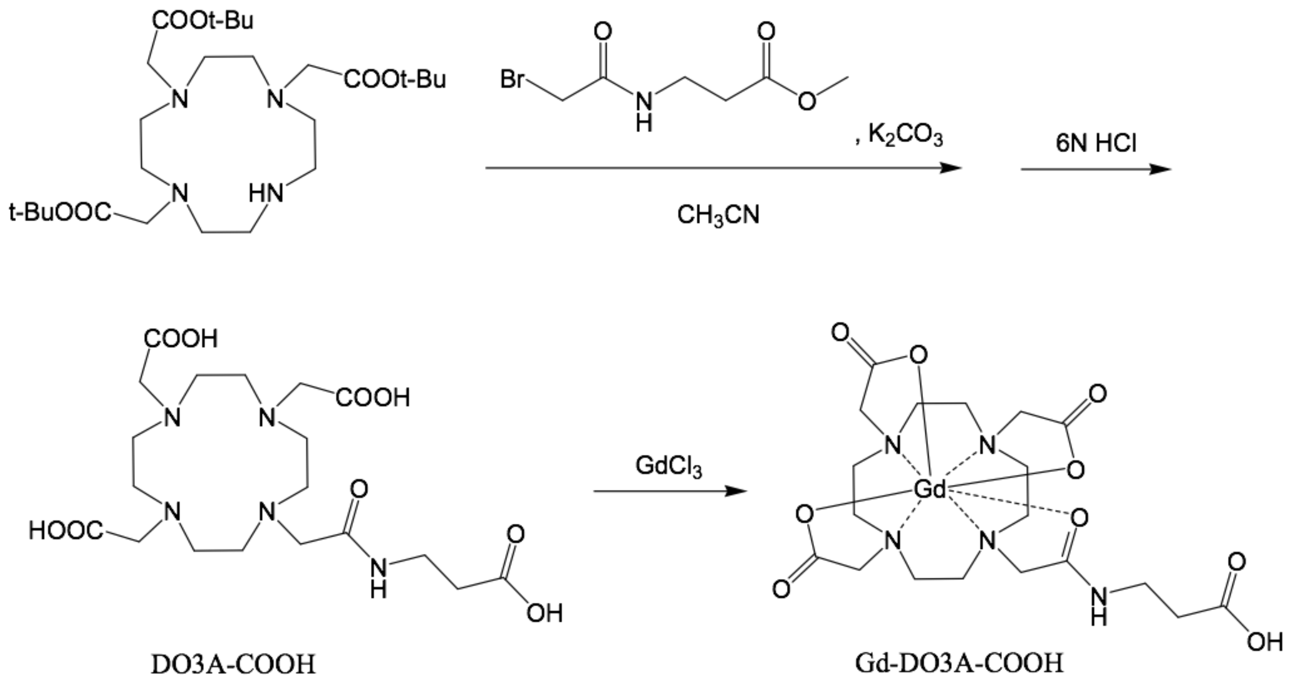

Scheme. Synthetic scheme of Gd-DO3A-COOH.

Figure A

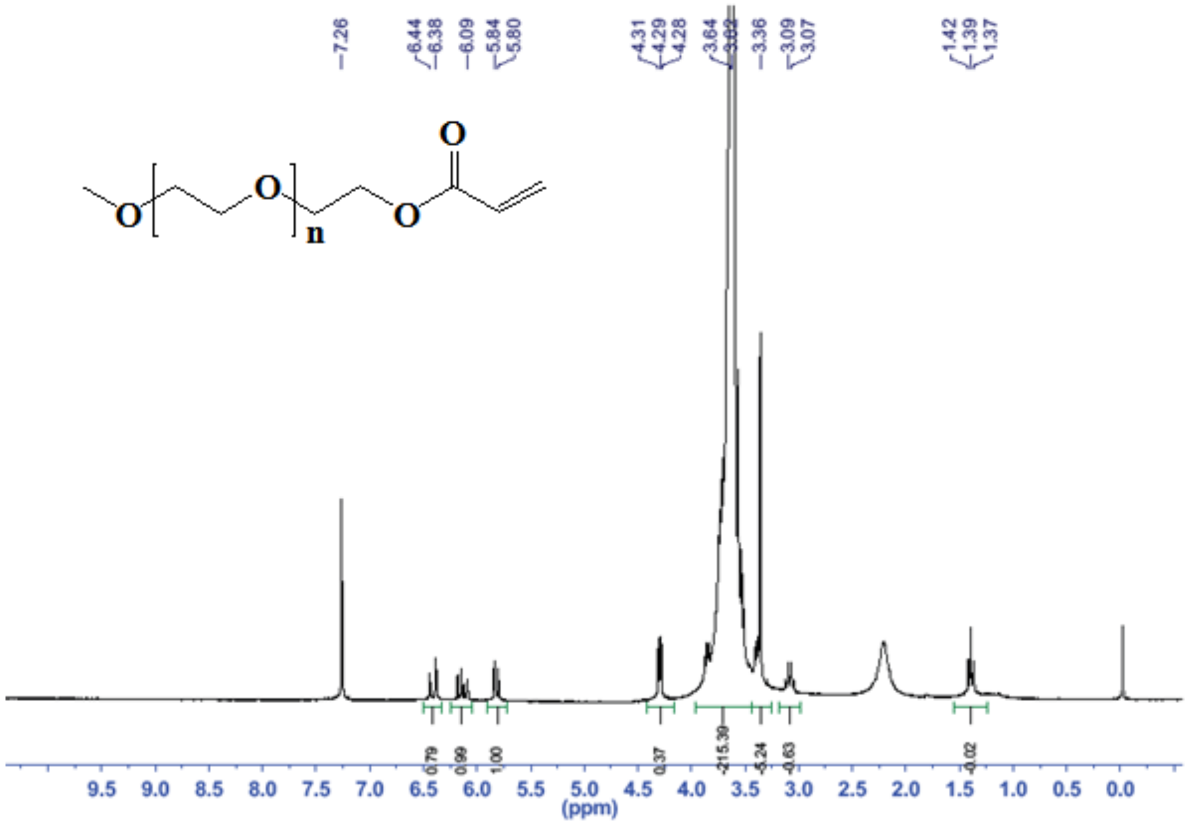

Figure A. ^{1}H NMR spectrum of mPEG-Ac.

Figure B

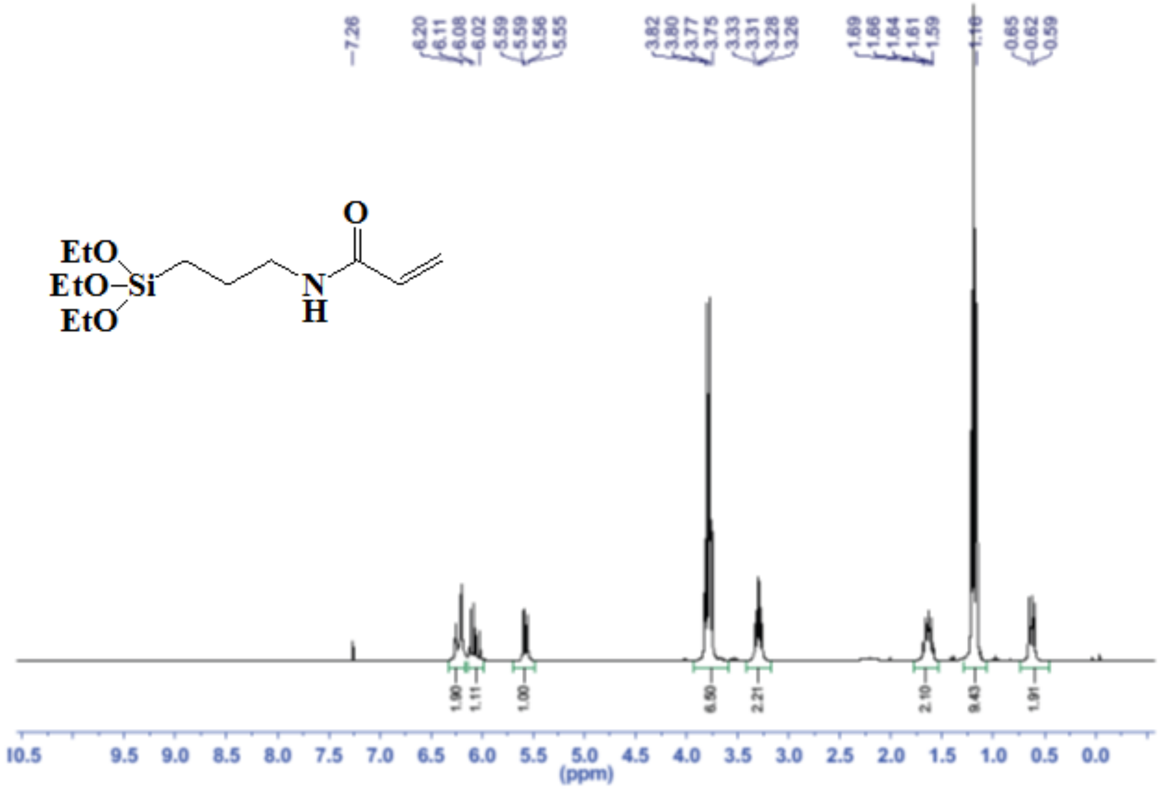

Figure B. ^{1}H NMR spectrum of silane-Ac.

Figure C

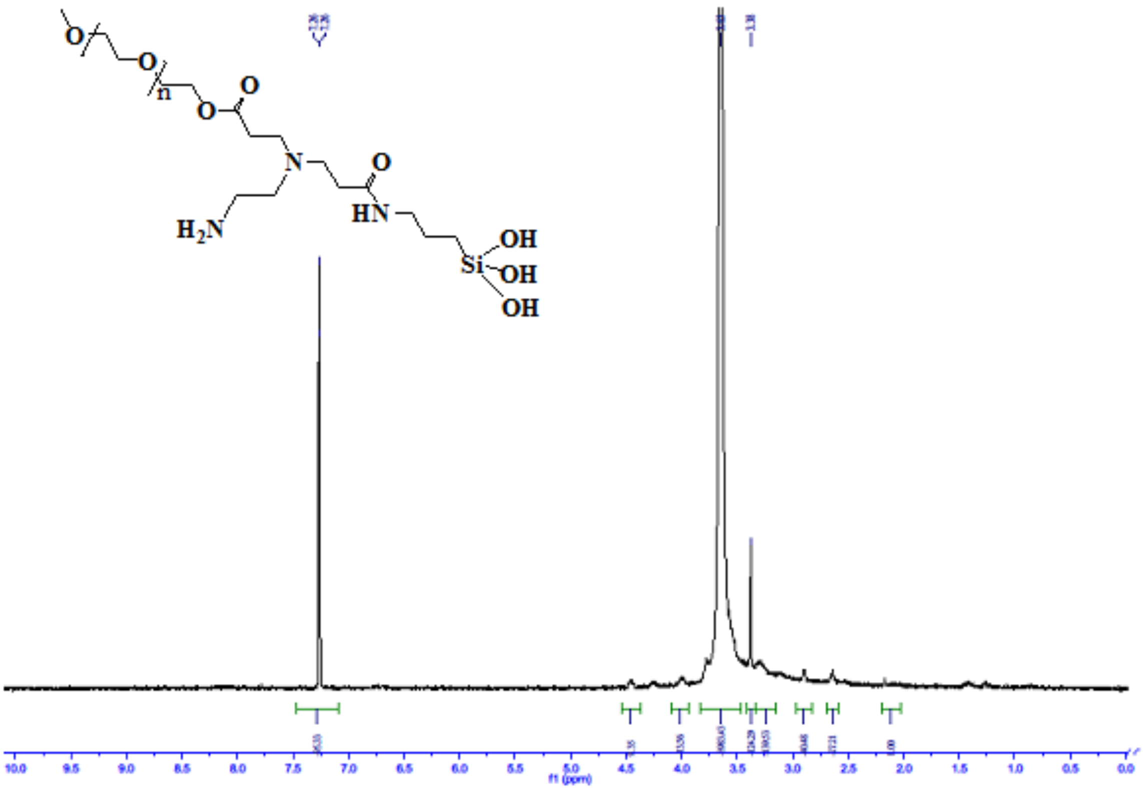

Figure C. ^{1}H NMR spectrum of mPEG-Nboc-silane.

Figure D

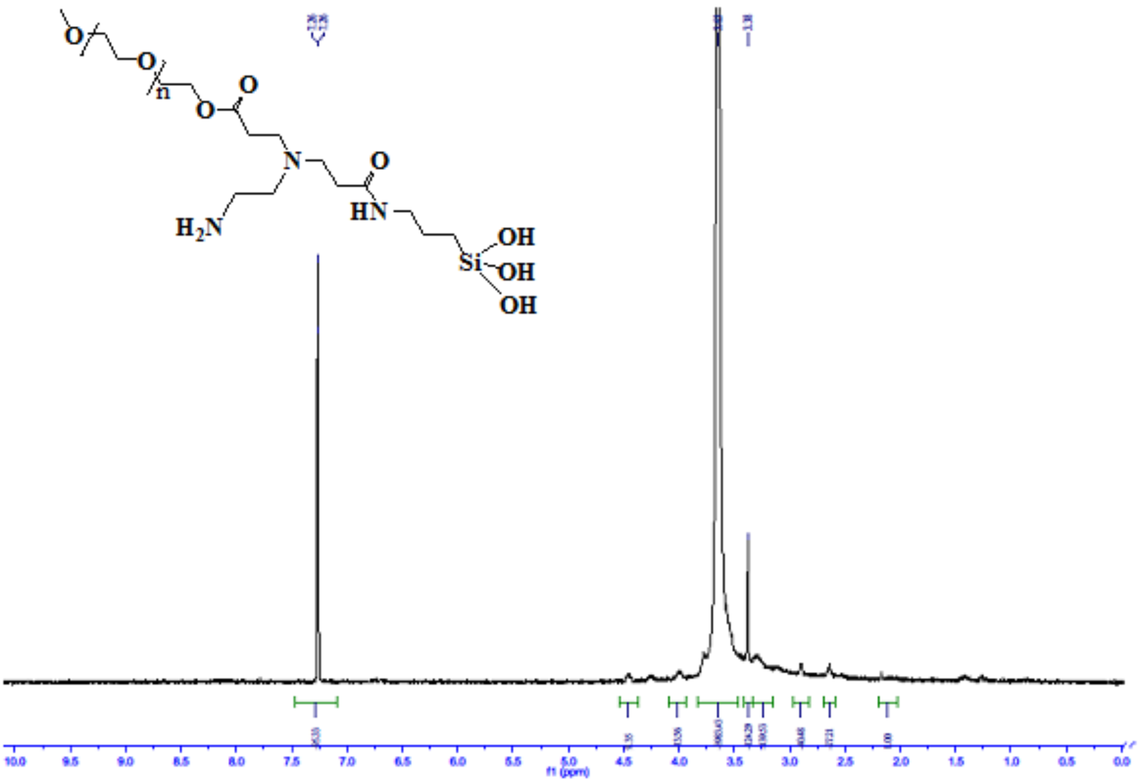

Figure D ^{1}H NMR spectrum of mPEG-NH_2-silane.

Figure E

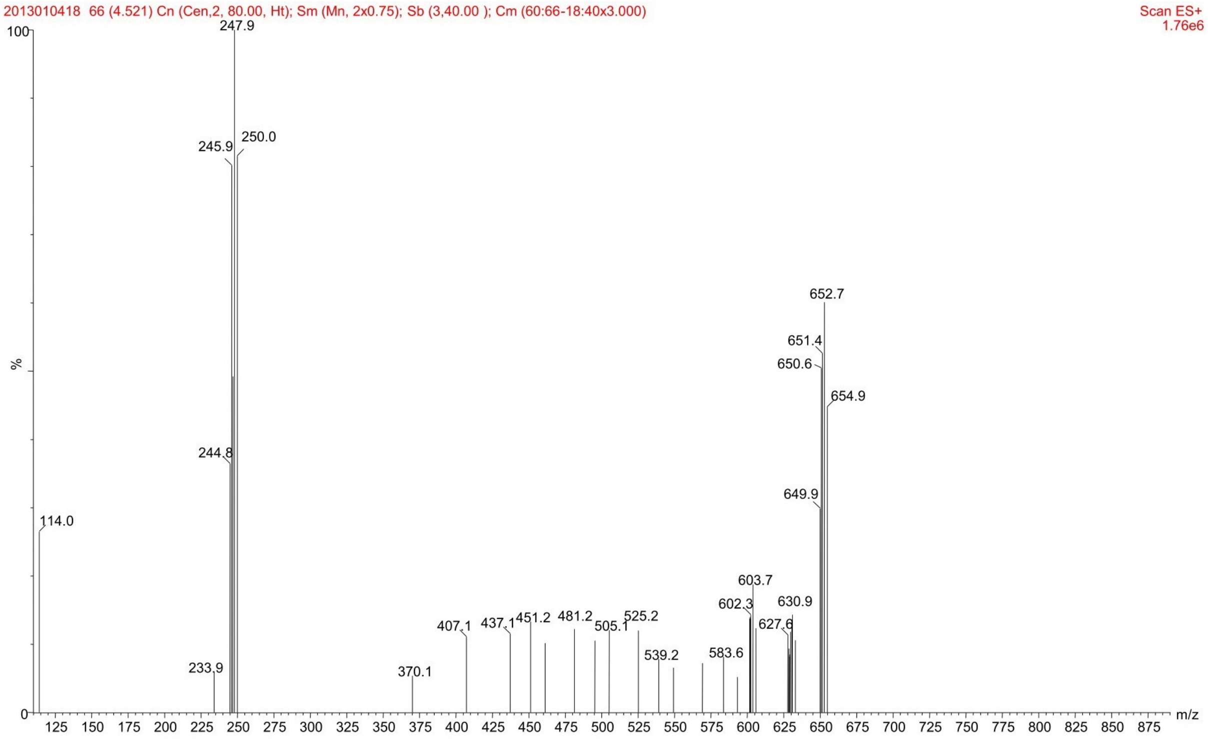

Figure E. ESI-MS spectrum of Gd-DO3A-COOH.

Figure F

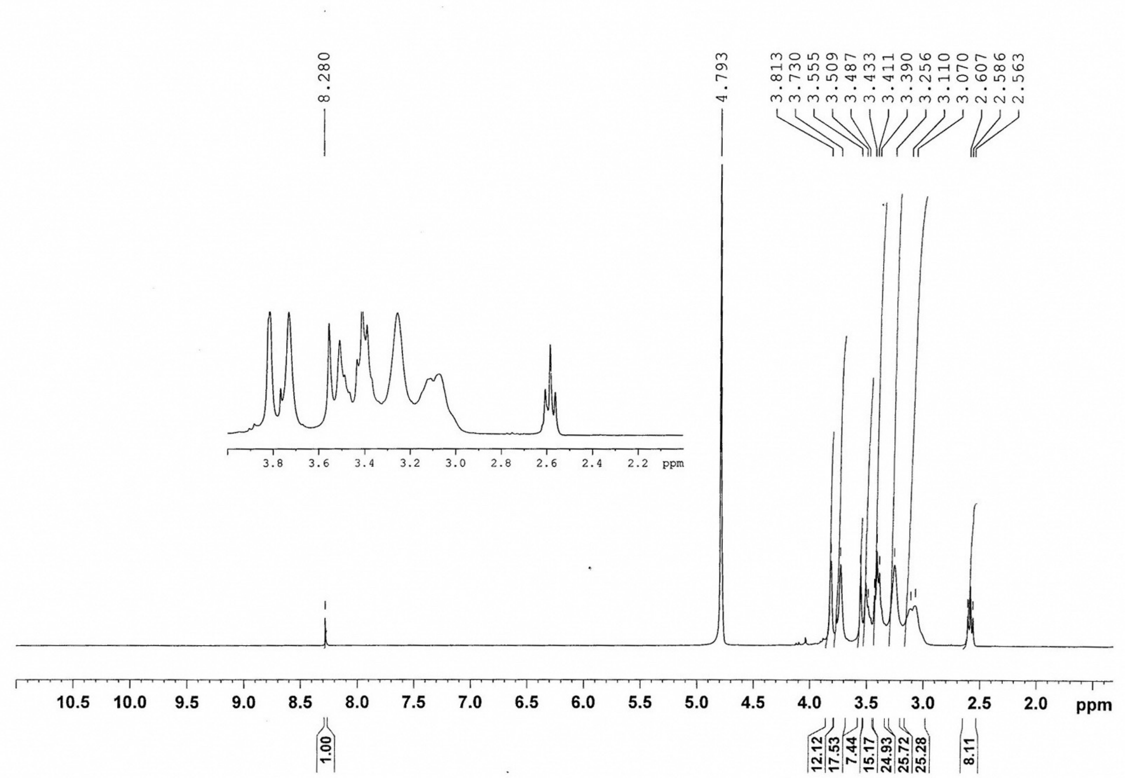

Figure F. ^{1}H NMR spectrum of 1-(methyl-3-acetamidopropanoate)-4,7,10-tris(acetic acid)-1,4,7, 10-tetraazacyclodecane (DO3A-COOH).

Figure G

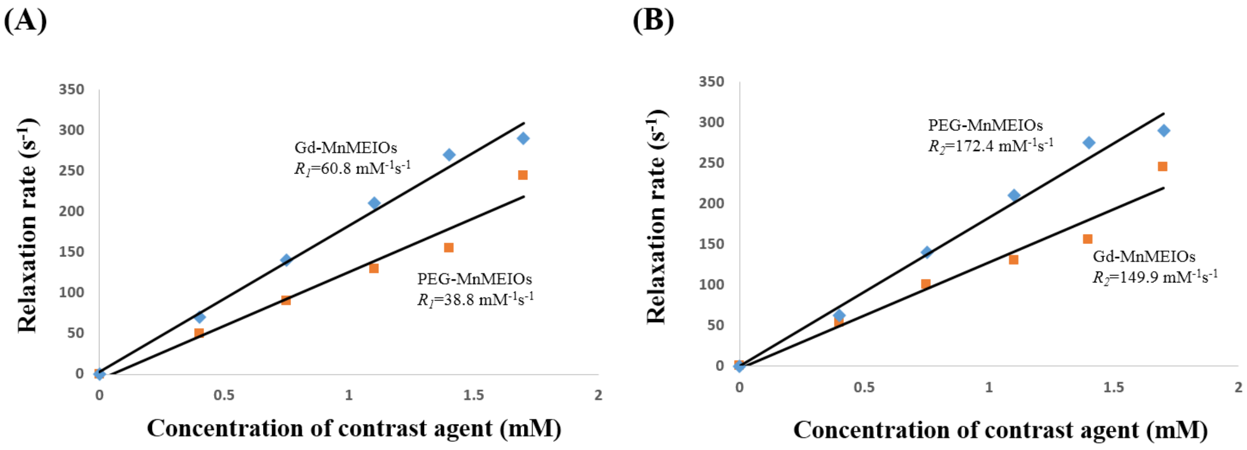

Figure G. The r_1 and r_2 of PEG-MnMEIO and Gd-MnMEIO in 20 MHz at 37.0 ± 0.1°C.
